# Phytochemical analysis and evaluation of the antibacterial and antibiotic potentiation activities of the aqueous extract of *Cordia oncocalyx* Allemão (Boraginaceae)

**DOI:** 10.1016/j.pscia.2024.100042

**Published:** 2024-05-18

**Authors:** José Thyalisson da Costa Silva, José Jailson Lima Bezerra, Talysson Felismino Moura, Rafael Pereira da Cruz, Maraiza Gregorio de Oliveira, Adrielle Rodrigues Costa, Felicidade Caroline Rodrigues, João Arthur de Oliveira Borges, Terezinha Raila Ramos de Sousa, Maria Flaviana Bezerra Morais-Braga, Henrique Douglas Melo Coutinho, José Weverton Almeida-Bezerra

**Affiliations:** a Departamento de Ciências Biológicas, Universidade Regional do Cariri – URCA, 63105-000, Crato, Brazil; b Departamento de Botânica, Universidade Federal de Pernambuco – UFPE, 50670-901, Recife, Brazil; c Centro de Ciências Agrárias e Biodiversidade, Universidade Federal do Cariri - UFCA, 63133-610, Crato, CE, Brazil; d Departamento de Química Biológica, Universidade Regional do Cariri – URCA, 63105-000, Crato, Brazil

**Keywords:** *Auxemma oncocalyx*, Multidrug-resistant microorganisms, Natural products, Flavonoids, Antimicrobial

## Abstract

The global antibiotic resistance crisis highlights the inappropriate use of medicines by the population and the lack of development of new antimicrobial agents. According to various studies, natural products are promising alternatives for combating bacterial resistance and treating infectious diseases. Accordingly, the present study aimed to analyze the chemical composition and evaluate the antibacterial and antibiotic potential of an aqueous extract of *Cordia oncocalyx* Allemão (AECO). Phytochemical analyses were performed using high-performance liquid chromatography equipped with a diode array detector (HPLC-DAD). The minimum inhibitory concentration (MIC) was used to evaluate the antibacterial activity of *C. oncocalyx* against conventional and multidrug-resistant (MDR) bacterial strains (*Escherichia coli, Pseudomonas aeruginosa*, and *Staphylococcus aureus*). According to HPLC-DAD analysis, the following compounds could be identified in the aqueous extract of *C. oncocalyx*: luteolin (3.07 ± 0.04 mg/g), caffeic acid (1.05 ± 0.03 mg/g), ellagic acid (0.62 ± 0.05 mg/g), and quercetin (0.58 ± 0.01). AECO did not exhibit antibacterial activity when administered alone (MIC >512 μg/mL). However, when combined with gentamicin, ampicillin, and norfloxacin, AECO potentiated the action of these antibiotics against the multi-resistant strains of *P. aeruginosa* and *S. aureus*. Although clinical relevance was not revealed by the *in vitro* tests against pathogenic bacteria, AECO can combined with commercial antibiotics to improve their antibacterial effects. Future studies focusing on the mechanisms of action of the compounds isolated from *C. oncocalyx* and toxicological tests are fundamental.

## Graphical abstract

**Figure:**
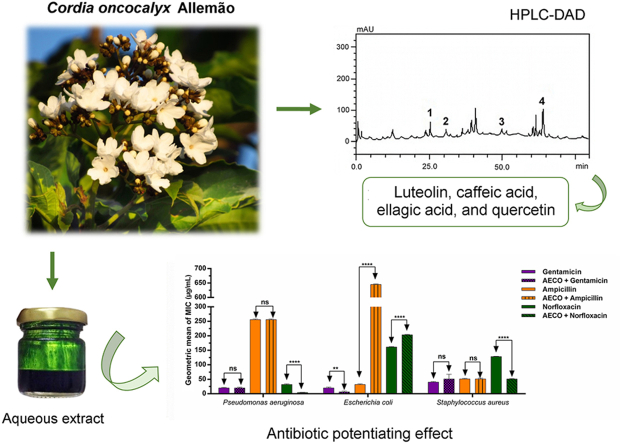

## Abbreviations

AECO: Aqueous extract of *Cordia oncocalyx*
ANOVA: Analysis of variance
ATCC: American Type Culture Collection
BHI: Brain heart infusion
DNA: Deoxyribonucleic acid
FTIR: Fourier transform infrared spectroscopy
HCDAL: Herbarium Caririense Dárdano de Andrade-Lima
HPLC-DAD: High-performance liquid chromatography with diode array detector
LCMS: Liquid chromatography coupled to mass spectrometry
MIC: Minimum inhibitory concentration
MDR: Multidrug-resistant
NMR: Nuclear Magnetic Resonance
NS: Not significant
RT: Retention time;
SD: Standard deviation
URCA: Universidade Regional do Cariri

## 1 Introduction

Following the discovery of penicillin by Alexander Fleming in 1928, antibiotics, such as aminoglycosides, tetracyclines, glycopeptides, cephalosporins, and triazoles, have been widely used to treat infectious diseases [[1], [2], [3]]. However, bacterial resistance has intensified over time and has become a serious public health problem due to the inappropriate use of antibiotics by the population [[4], [5], [6]]. Considering the urgency in the search for new effective antimicrobials, natural products alone or combined with commercial drugs represent a viable alternative for combating various strains of multi-resistant bacteria that negatively affect human and animal health [[7], [8], [9], [10]].

The genus, *Cordia*, belongs to the Boraginaceae family and contains more than 300 species that are widely distributed in various tropical regions worldwide [11]. Of these, 58 species are found in Brazil and 30 are considered endemic [12]. Based on ethnobotanical surveys, some representatives of this genus, such as *Cordia myxa* L. [13], *Cordia platythyrsa* Bak. [14], *Cordia dichotoma* G. Forst. [15], and *Cordia africana* Lam [16], are widely used in traditional medicine to treat inflammation and infectious diseases caused by pathogenic microorganisms. *Cordia oncocalyx* Allemão (synonym: *Auxemma oncocalyx* Taub.) is known as “louro-branco,” “pau-branco,” “freijó,” or “pau-branco-preto” in the Northeast region of Brazil, and the infusion or decoction of its aerial parts are traditionally used against external ulcers and wound healing [17].

Previously, natural products obtained from species of the genus, *Cordia*, were reported to display promising pharmacological potential, specifically antifungal, antibacterial, and antiparasitic activities [[18], [19], [20]]. Oncocalyxone A, isolated from *C. oncocalix*, has been the target of several scientific investigations owing to its broad pharmacological spectrum in *in vitro* and *in vivo* assays [[21], [22], [23], [24]]. Among the main properties of this benzoquinone, its antileukemic [22], anti-inflammatory, analgesic, and antimicrobial activities are often highlighted [23]. According to Silva et al. [24], Oncocalyxone A exhibited antibacterial and antibiofilm potential against several strains of gram-positive and gram-negative bacteria, but did not demonstrate antifungal potential.

Considering the previously reported promising antibacterial effects of this plant and the need to identify efficient natural products against multi-resistant disease-causing microorganisms, a phytochemical analysis of the aqueous extract of *C. oncocalyx* was performed in the present study and the potentiating effect of its combination with commercial antibiotics against pathogenic bacteria of public health interest was determined.

## 2 Methodology

### 2.1 Plant material

**Fig. 1:**
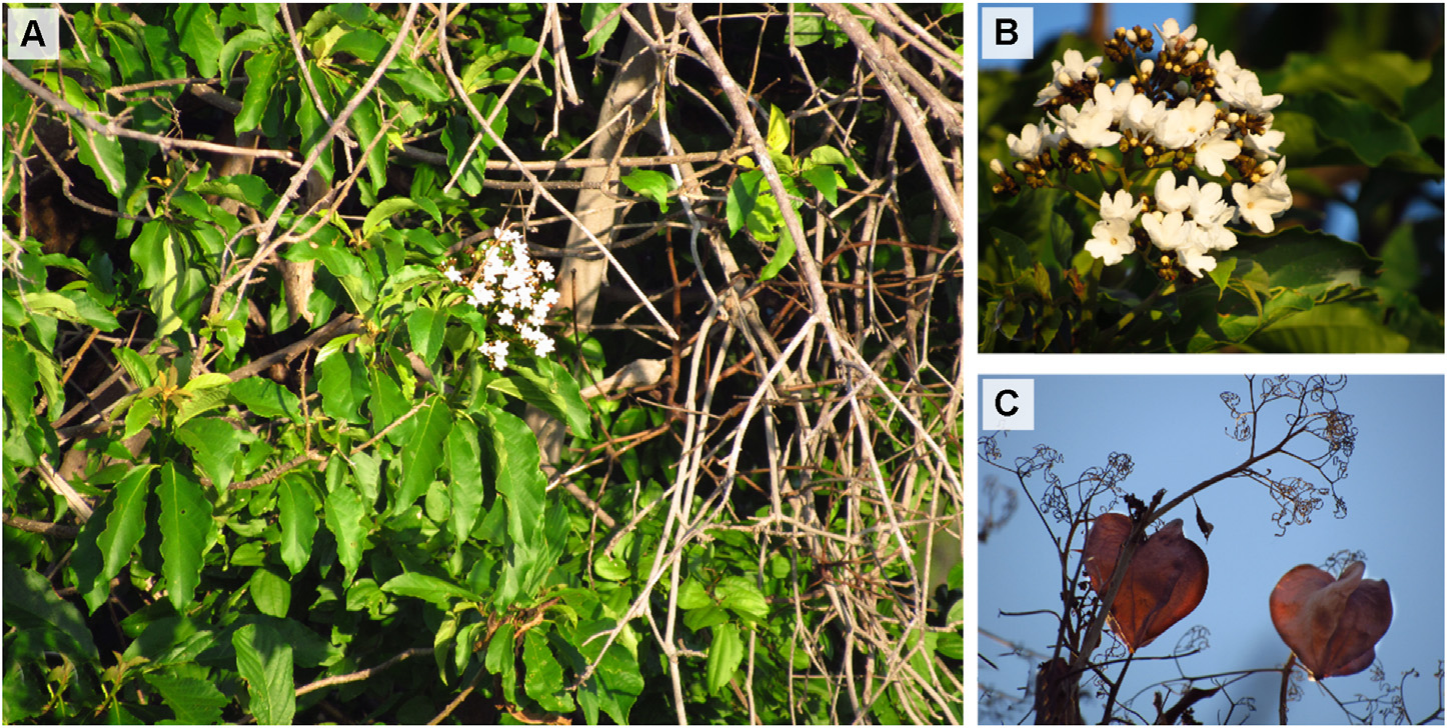
*Cordia oncocalyx* identified in the municipality of Crato, Ceará, Brazil. A) General view of the plant. B) Inflorescence. C) Fruits.

The leaves of *Cordia oncocalyx* (Fig. 1) were collected from the municipality of Crato, Ceará, Brazil (geographic coordinates: −7.238420 S, −39.415600 W) in January 2016. One specimen was identified and herborized at the Herbarium Caririense Dárdano de Andrade-Lima (HCDAL), Universidade Regional do Cariri (URCA) (identification number: 12.784).

### 2.2 Plant extract

First, *C. oncocalyx* leaves were dehydrated and crushed. To prepare the aqueous extract of *Cordia oncocalyx* (AECO), 250 g of the leaves was placed in glass bottles with 2 L of boiling distilled water and incubated for 72 h. The resulting infusion was filtered twice through cotton. The aqueous extract was freeze-dried to remove the solvent and obtain the concentrated crude extract.

### 2.3 Phytochemical analysis

#### 2.3.1 Chemicals

Phosphoric, gallic, ellagic, chlorogenic, and caffeic acids were purchased from Merck® (Darmstadt, Germany). Luteolin, rutin, quercetin, and kaempferol were purchased from Sigma-Aldrich®. All chemicals used were of analytical grade.

#### 2.3.2 Analysis via high-performance liquid chromatography (HPLC-DAD)

Samples of the aqueous extract of *C. oncocalyx* (concentration, 10 mg/mL) were injected into an SIL-20A Shimadzu Auto sampler. Separations were carried out using a Phenomenex C_18_ column (4.6 mm × 250 mm x 5 μm particle size). The mobile phase comprised water with 1 % phosphoric acid (v/v) (solvent A) and HPLC grade methanol (solvent B). The flow rate was 0.6 mL/min and injection volume was 40 μL. The following gradient was employed: 5% solvent B, 15% solvent B at 10 min, 30% solvent B at 35 min, 65% solvent B at 50 min, 98% solvent B at 65 min, and isocratic elution from 70 min to 75 min. After 80 min, the gradient returned to the initial conditions, according to the method described by Adefegha et al. [25], with slight modifications.

The sample and mobile phase were filtered through a 0.45 μm membrane filter (Millipore) and then degassed via an ultrasonic bath prior to use. Stock solutions of the standard references were prepared at concentrations ranging from 0.030 to 0.500 mg/mL. Quantification was performed by integrating the peaks using the external standard method at 254 nm for gallic and ellagic acids, 327 nm for chlorogenic acid and caffeic acid, and 366 nm for luteolin, quercetin, rutin, and kaempferol. The chromatographic peaks were confirmed by comparing their retention times with those of the reference standards and DAD spectra (200–600 nm). All chromatographic operations were performed at ambient temperature and in triplicate.

### 2.4 Antibacterial activity

#### 2.4.1 Strains, culture media, and drugs

Antibacterial activity was determined based on the MIC of conventional and multi-resistant bacterial strains. The reference strains were *Escherichia coli* ATCC 25922, *Pseudomonas aeruginosa* ATCC 25853, and *Staphylococcus aureus* ATCC 25923, whereas the resistant strains were *E. coli* 06, *P. aeruginosa* 24, and *S. aureus* 10. Brain Heart Infusion (BHI; Merck KGaA, Darmstadt, Germany) was used as the culture medium, according to the manufacturer's instructions.

Bacterial strains were diluted in 3 mL of sterile saline (0.9% NaCl) and their turbidity was compared with the McFarland standard adjusted to 0.5 on the scale (equivalent to 1.5 × 10^8^ colony forming units/mL) [26]. Gentamicin, ampicillin, and norfloxacin were used as commercial antibiotics.

#### 2.4.2 Minimum inhibitory concentration (MIC)

To test the bacterial growth inhibition capacity of AECO, 100 μL of inoculum solution and 900 μL of culture medium (BHI) were dispensed in 96-well microplates. In addition, 0.5–512 μg/mL of AECO was also added to the wells. The microplates were incubated in a bacteriological oven at 37 °C for 24 h [27].

After incubation, liquid resazurin was used as the developing agent; this substance reacts through an oxidation-reduction process, indicating bacterial growth. After 1 h of reaction, the presence of a violet color (indicating no growth) or light pink color (indicating an increase in the presence of bacteria) was determined.

#### 2.4.3 Antibiotic potentiating effect

AECO was tested at sub-inhibitory concentrations of the matrix concentration (MIC/8) and combined with the commercial antibiotics, gentamicin, ampicillin, and norfloxacin. The tests were carried out via microdilution in wells containing 0.5–512 μg/mL of antibiotics (100 μL per well). The plates were incubated in a bacteriological oven at 37 °C for 24 h, and the experiments were repeated in triplicate [28].

### 2.5 Statistical analysis

The antibacterial activity of AECO was measured in triplicate. All statistical analyses were performed using GraphPad Prism software (version 6.0; GraphPad Software, Inc., San Diego, CA, USA). Data were analyzed using one-way ANOVA with Tukey's test.

## 3 Results

### 3.1 Analysis via high-performance liquid chromatography (HPLC-DAD)

Based on the analysis of the aqueous extract of *C. oncocalyx* using HPLC-DAD, four phenolic and flavonoid phytochemicals were identified (Fig. 1). The main compounds in AECO were luteolin (3.07 ± 0.04 mg/g) and caffeic acid (1.05 ± 0.03 mg/g). Ellagic acid (0.62 ± 0.05 mg/g) and quercetin (0.58 ± 0.01 mg/g) were also identified in AECO (Table 1).

**Table 1:** Quantification of the compounds in the *Cordia oncocalyx* aqueous extract identified using high-performance liquid chromatography (HPLC-DAD).

Peaks	Compounds	Amount (mg/g)
1	Caffeic acid	1.05 ± 0.03
2	Ellagic acid	0.62 ± 0.05
3	Quercetin	0.58 ± 0.01
4	Luteolin	3.07 ± 0.04

The results are expressed as mean ± standard deviation (SD) of three determinations.

### 3.2 Antibacterial activity

To determine the presence of control activity in strains of *S. aureus, P. aeruginosa*, and *E. coli*, the MIC was evaluated for standard and multidrug-resistant strains, as shown in Table 2. Notably, a minimum clinically relevant inhibitory concentration was not found for AECO as its possible action exceeds the highest concentration evaluated (MIC >512 μg/mL).

**Table 2:** Minimum Inhibitory Concentration (MIC, μg/mL) of the aqueous extract of *Cordia oncocalyx* against different strains of pathogenic bacteria.

Strains	MIC (μg/mL)		
	*P. aeruginosa*	*E. coli*	*S. aureus*
Standard strains (ATCC)	>512	>512	>512
Multidrug-resistant	>512	>512	>512

The standard strains were *Pseudomonas aeruginosa* ATCC 25853, *Escherichia coli* ATCC 25922, and *Staphylococcus aureus* ATCC 25923 while the multidrug resistance strains were *Pseudomonas aeruginosa* 24, *Escherichia coli* 06, and *Staphylococcus aureus* 10.

### 3.3 Antibiotic potentiating effect

How AECO affects the action of antibiotics by potentiating or interfering with their activity was assessed. A combination experiment (AECO ​+ ​antibiotics) was also performed. AECO exhibited different actions when combined with gentamicin, ampicillin, and norfloxacin; these actions varied from synergistic, indifferent, and antagonistic, as shown in Fig. 3. AECO exhibited a potentiating effect when combined with norfloxacin, which inhibited bacterial DNA synthesis in strains of *P. aeruginosa* (gram-negative) and *S. aureus* (gram-positive), ultimately reducing the MIC of the antibiotic.

**Fig. 2:**
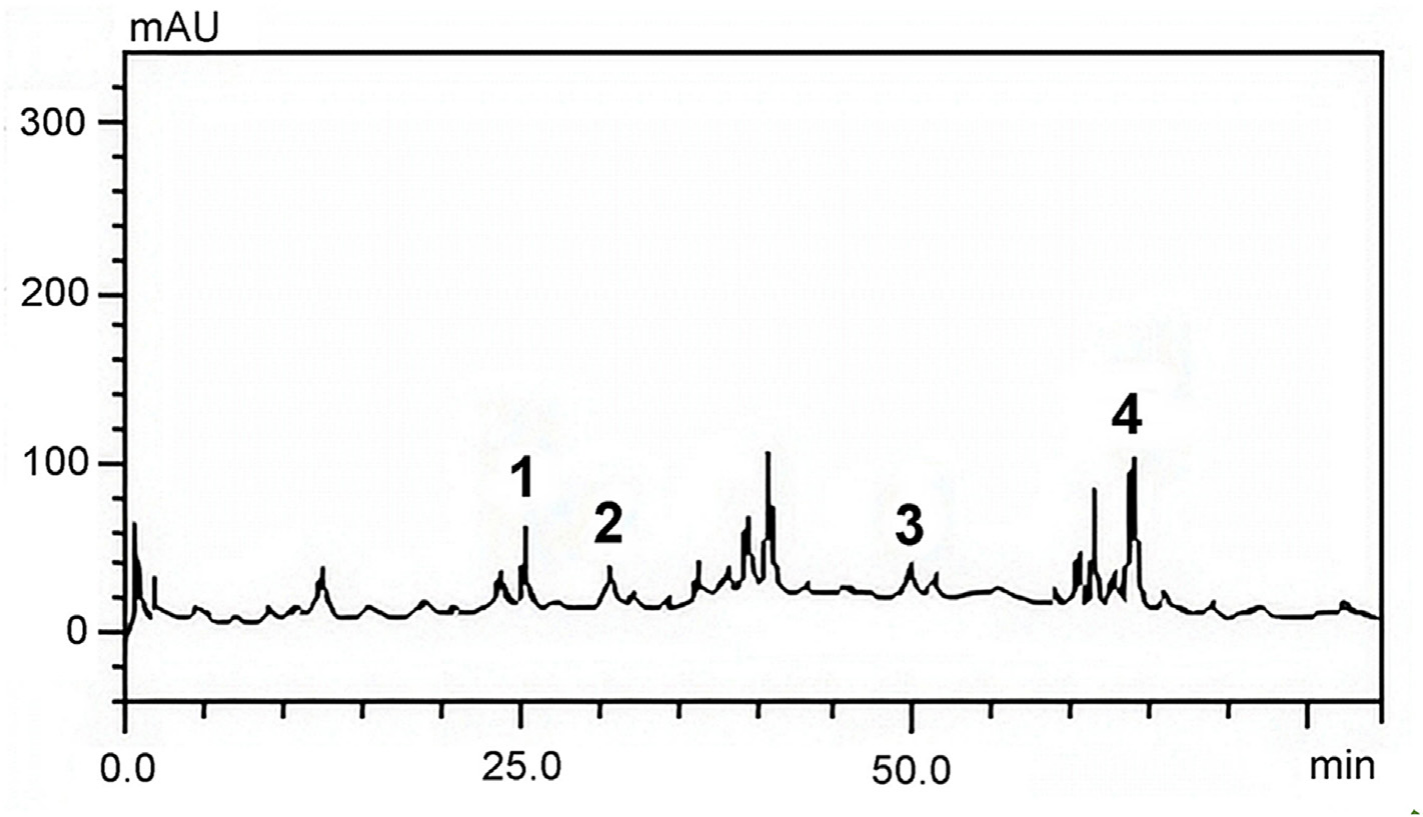
Representative high-performance liquid chromatography profile of the aqueous extract of *Cordia oncocalyx*. Caffeic acid (Rt: 25.04 min, peak 1), ellagic acid (Rt: 31.97 min, peak 2), quercetin (Rt: 49.07 min, peak 3), and luteolin (Rt: 64.35 min, peak 4). Rt: retention time.

**Fig. 3:**
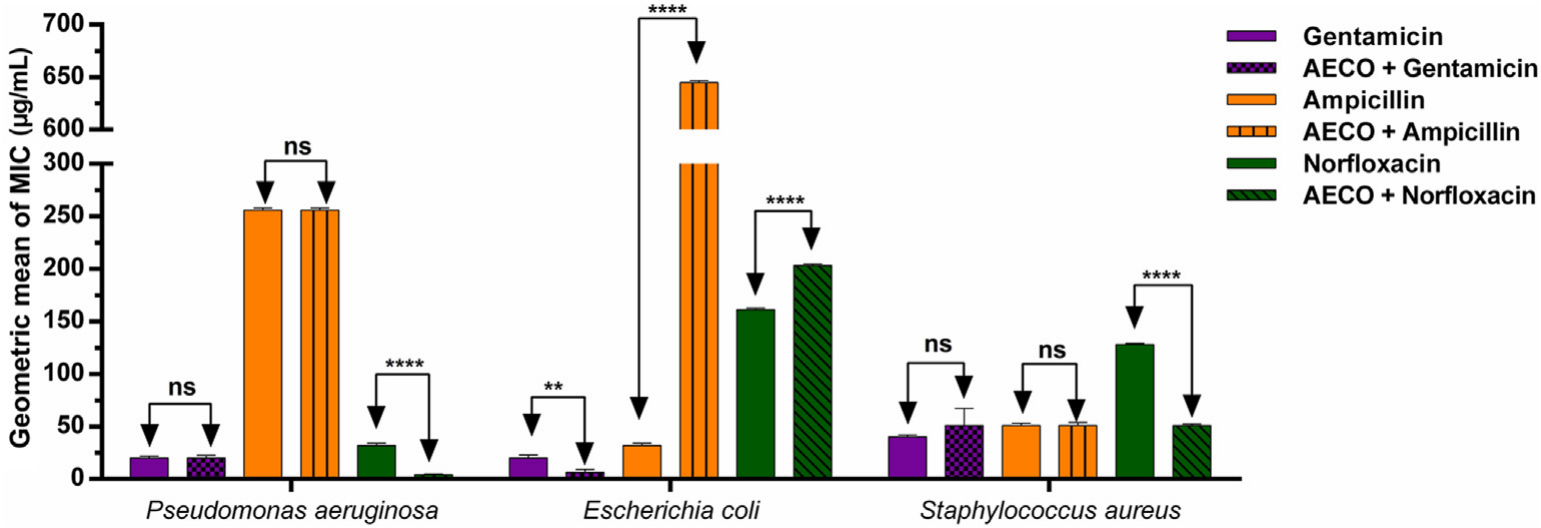
Minimum inhibitory concentration (MIC) of the aqueous extract of *Cordia oncocalyx* associated with conventional antibiotics against multidrug-resistant bacteria. AECO: aqueous extract of *Cordia oncocalyx*. ns: not significant (P ​> ​0.05), ∗∗∗∗: P ​< ​0.0001, and ∗∗: P ​< ​0.001. Bars indicate the standard deviation (n ​= ​3).

AECO increased the effectiveness of gentamicin, which belongs to the aminoglycoside group that directly inhibits protein synthesis, against *E. coli* (gram-negative). However, an adverse interaction was observed between the extract and ampicillin, and the extract and norfloxacin against *E. coli*.

## 4 Discussion

In this study, the antibacterial activity of AECO, which modifies the action of conventional antibiotics, was evaluated to elucidate its potential to treat and control different pathogenic bacteria, including gram-positive and gram-negative bacteria. The potentiating action of antibiotics is often highlighted as their improved and combined actions become an alternative complementary measure to treat diseases caused by multidrug-resistant (MDR) microorganisms, ultimately enabling the identification of new promising therapeutic alternatives [29,30].

Silva et al. [31] identified caffeic acid, ellagic acid, quercetin, and luteolin in the aqueous extract of *C. oncocalyx*, thereby corroborating our findings. The positive relationship between flavonoids, quercetin, and luteolin, and antibiotics is promising owing to the significant synergism. Evaluation of this action against isolated strains of *S. aureus* revealed the potential action caused by changes in the cell wall and bacterial cell membrane, which increases and allows antibiotic entry [[32], [33], [34]]. In addition, biofilms are disturbed and bacterial communication is disrupted [35]. Therefore, the flavonoids in AECO may also be directly involved in the potentiating effects of the antibiotics, gentamicin, ampicillin, and norfloxacin (Fig. 3).

The antimicrobial activity of quercetin is related to the disruption of cell membrane integrity, inhibition of nucleic acid synthesis, inhibition of biofilm formation, mitochondrial dysfunction, and inhibition of pathogen virulence factor expression [36]. According to Qian et al. [37], luteolin harms bacterial cell membranes, induces cellular morphological changes, and inhibits biofilm formation. Therefore, luteolin is a promising alternative to commercial antibiotics or adjuvants in conventional antibiotic therapies [38].

Caffeic acid, another compound identified in AECO, also exhibits promising antimicrobial activity. According to Khan et al. [39], this phenolic compound alters membrane permeability, inhibits enzymatic activity, and damages the DNA of bacterial and fungal pathogens. Furthermore, Kępa et al. [40] found that the combination of caffeic acid with the antibiotics, erythromycin, clindamycin, cefoxitin, and vancomycin, induced promising synergistic activity against strains of *S. aureus* isolated from infectious wounds, reinforcing the hypothesis that natural products can enhance the effect of commercial antibiotics against resistant microorganisms.

In addition to the compounds identified in AECO via HPLC-DAD (Fig. 2), other constituents of *C. oncocalyx* may be involved in the observed antibacterial activity. For example, the benzoquinone, oncocalyxone A, is reported to be the main chemical compound isolated from *C. oncocalyx* [21]. According to Silva et al. [31], this compound has antibacterial and antibiofilm potential against several gram-positive and gram-negative bacteria. Therefore, the effects of oncocalyxone A, isolated from *C. oncocalyx* combined with commercial antibiotics against multidrug-resistant bacteria that cause serious infections in humans and animals must be evaluated.

## 5 Conclusion

The aqueous extract of *C. oncocalyx* leaves contains flavonoids and phenolic compounds of pharmacological interest, such as luteolin, caffeic acid, ellagic acid, and quercetin. AECO did not display any clinical relevance against pathogenic bacterial strains when administered alone. However, when combined with the antibiotics, norfloxacin, ampicillin, and gentamicin, AECO potentiated their effects against the multi-resistant strains of *P. aeruginosa* and *S. aureus*. These findings encourage further investigations into the synergy between natural products and commercial antibiotics. Furthermore, future studies should aim to elucidate the mechanisms of action of compounds isolated from *C. oncocalyx* against resistant bacterial strains, and evaluate the biological safety of this plant through *in vitro* and *in vivo* toxicological tests. New analyses using liquid chromatography coupled with mass spectrometry (LC-MS), fourier transform infrared spectroscopy (FTIR), and nuclear magnetic resonance (NMR) are also necessary to identify other compounds in the aqueous extract of this plant.

## Data availability

Dataset is available on request from the authors.

## Ethics approval

Not applicable.

## Funding information

This research did not receive any specific grant from funding agencies in the public, commercial, or not-for-profit sectors.

## CRediT authorship contribution statement

**José Thyalisson da Costa Silva:** Writing – review & editing, Writing – original draft, Project administration, Conceptualization. **José Jailson Lima Bezerra:** Writing – review & editing, Formal analysis. **Talysson Felismino Moura:** Investigation. **Rafael Pereira da Cruz:** Methodology. **Maraiza Gregorio de Oliveira:** Methodology. **Adrielle Rodrigues Costa:** Investigation. **Felicidade Caroline Rodrigues:** Methodology. **João Arthur de Oliveira Borges:** Software. **Terezinha Raila Ramos de Sousa:** Investigation. **Maria Flaviana Bezerra Morais-Braga:** Supervision. **Henrique Douglas Melo Coutinho:** Supervision. **José Weverton Almeida-Bezerra:** Writing – review & editing, Writing – original draft, Project administration, Conceptualization.

All the authors have read and approved the final version of this manuscript.

## Declaration of competing interests

The authors declare that they have no known competing financial interests or personal relationships that could have appeared to influence the work reported in this paper.
